# Mechanistic insights into SLAMF8-mediated prostate cancer metastasis via the TLR4-NF-κB pathway

**DOI:** 10.1186/s12967-025-07234-3

**Published:** 2025-10-29

**Authors:** Qiang Su, Zhao Li, Ning Zhang, Wei Mu, Yi Hu, Bin Dai

**Affiliations:** 1 Department of Clinical Laboratory Medicine, Yuquan Hospital, School of Clinical Medicine, Tsinghua University, Beijing, 100040 China; 2 Department of Pathology, Beijing Shijitan Hospital, Capital Medical University, Beijing, 100038 China; 3 MRI Department, Beijing Shijitan Hospital, Capital Medical University, Beijing, 100038 China; 4 School of Engineering Medicine, Beihang University, Beijing, 100191 China; 5 Key Laboratory of Big Data-Based Precision Medicine (Beihang University), Ministry of Industry and Information Technology, Beijing, 100191 People’s Republic of China; 6 Biomedical Innovation Center, Beijing Shijitan Hospital, Capital Medical University, Beijing, 100038 China; 7 Neurosurgery Department, Beijing Shijitan Hospital, Capital Medical University, Beijing, 100038 China

**Keywords:** Prostate cancer, Metastasis, Immune checkpoint, SLAMF8, TLR4-NF-κB pathway

## Abstract

**Background:**

SLAMF8 functions as a cancer-promoting immune checkpoint and could be targeted for therapy in multiple cancer types. Its effect on the immune microenvironment and metastasis of prostate cancer (PCa) is not well understood.

**Method:**

We analyzed SLAMF8 distribution in PCa and normal tissues using TIMER and examined its role in drug sensitivity and immunotherapy for PCa. The prognostic value and clinical relevance of SLAMF8 in PCa were assessed using multiple datasets. GO, KEGG, and GSEA analyses identified dysregulated pathways in tumors with varying SLAMF8 levels. Tumor purity and immune cell infiltration, and their correlation with SLAMF8 overexpression, were analyzed and confirmed in PCa samples. Additionally, CCK-8, flow cytometry, and transwell assays evaluated the viability, apoptosis, and invasion capacity of SLAMF8-overexpressing PCa cells. Allograft models in C57BL/6 mice were used to study the effects of SLAMF8 overexpression on tumor growth and immune cell infiltration.

**Results:**

Compared to non-tumor tissues, SLAMF8 was overexpressed in PCa tumors. High SLAMF8 levels are linked to poor distant metastasis-free survival (DMFS), higher Gleason scores (GS), and advanced T stage. SLAMF8 expression in PCa tumors negatively correlates with tumor purity but positively correlates with the infiltration of B cells, T cells, dendritic cells, and macrophages. SLAMF8 overexpression in prostate cancer cells promoted cell growth, lowered apoptosis rates, and boosted invasion in vitro, alongside hastening tumor development in mice. This study demonstrates that SLAMF8 enhances PCa metastasis via the TLR4-NF-κB pathway. SLAMF8 is a potential predictor of distant metastasis and a promising target for PCa immunotherapy.

**Supplementary Information:**

The online version contains supplementary material available at 10.1186/s12967-025-07234-3.

## Background

Prostate cancer (PCa) is a highly common type of malignant tumor. PCa accounted for 14.2% of all new male cancers worldwide, and was responsible for 7.3% of male cancer deaths [1]. Prostate cancer in China has an estimated incidence of 134,200 and an estimated mortality of 47,500, ranking sixth and seventh respectively [2]. The epidemiological investigation in the United States in 2025 shows that the projected newly added cases of prostate cancer account for 30% of all male tumors, ranking the first, and the projected mortality rate accounts for 11% of all male tumors, ranking the second [3]. Most early PCa can be effectively treated with a comprehensive treatment plan. Existing standard therapies, however, lack efficacy for advanced PCa due to tumor aggressiveness, resistance to treatments, recurrences, and metastases. Only 30% of men survive 5 years after being diagnosed with metastatic PCa [4]. Thus, understanding the underlying mechanisms of PCa metastasis is crucial to developing novel therapies.

Recent studies have demonstrated that immune escape of tumor cells and abnormal immune surveillance influence cancer initiation, progression, and metastasis [5]. In PCa patients, prognosis and immunotherapy outcomes are significantly influenced by the tumor microenvironment (TME). PCa development is risked by intraprostatic inflammation [6]. Signaling lymphocytic activation molecule family member 8 (SLAMF8), as a SLAM family member, contributes importantly to immune and inflammatory activities [7]. It has been established that lymphocyte infiltration and high levels of immune-related genes, such as SLAMF8 and TNF, are related to a poor prognosis in breast cancer patients who are postmenopausal [8]. There is growing evidence that various SLAMF members act as independent prognostic indicators in cancers. For instance, SLAMF1/CD150 is differentially expressed in various hematologic cancers, including chronic lymphocytic leukemia (CLL) and multiple myeloma, suggesting its potential as a prognostic marker [9]. According to a study, gastric cancer serum samples showed elevated levels of SLAMF8, suggesting that it could serve as a biomarker for early detection and prognosis [10]. Anti-PD1 immunotherapy may be more effective for gastrointestinal tumors when SLAMF8 expression is high [11]. More studies are essential to explore the function of SLAMF8 in PCa metastasis.

The Toll-like Receptor (TLR) 4 gene polymorphism may be associated with PCa in Korean men [12]. There is evidence that recurrence of PCa is related to the expression of TLRs [13]. Two studies have discovered a connection between the NF-κB signaling pathway and bone metastasis in prostate cancer [14, 15]. SLAMF8 deletion has a significant inhibitory effect on TLR4 and NF-κB pathways [16]. In glioma, SLAMF8 may contribute to malignant progression, poor prognosis, and chemotherapy resistance through Toll-like receptors [17]. According to our previous study, SLAMF8 is an important marker for prostate cancer metastasis [18]. However, it is unknown whether SLAMF8 alters the immune microenvironment via the Toll-like receptor pathway to affect PCa progression. Further study of SLAMF8-TLRs-NF-κB in the biology of PCa would be beneficial. Moreover, the growing understanding of immune checkpoint interactions and combination therapies, as highlighted in recent meta-analytical perspectives and molecular reviews, provides a critical framework for evaluating novel targets like SLAMF8 [11, 19].

## Materials and methods

**Fig. 1:**
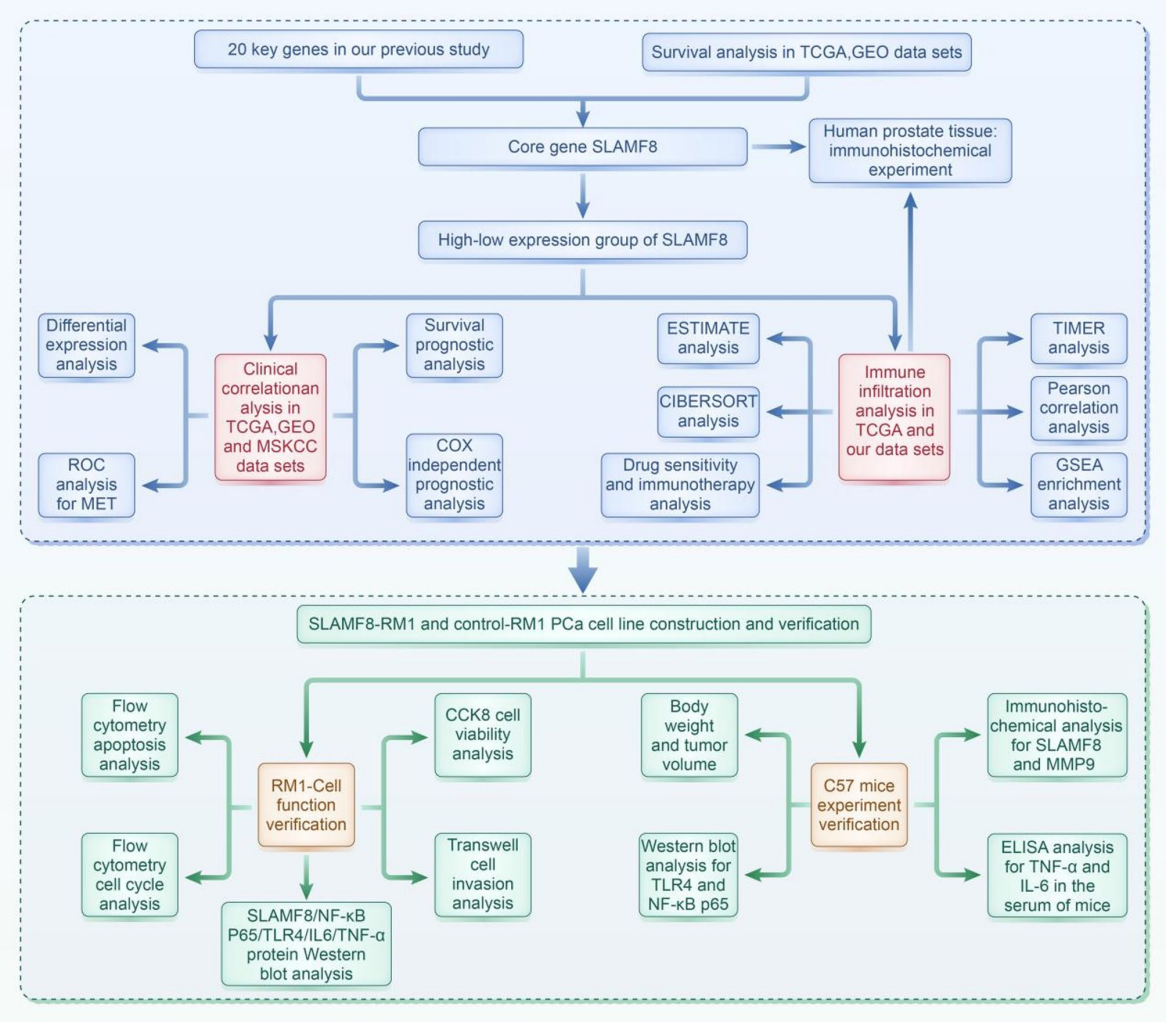
Flowchart of the study method

To validate our hypothesis, we performed bioinformatics analysis using datasets from the Cancer Genome Atlas (TCGA), Memorial Sloan Kettering Cancer Center (MSKCC), and the Gene Expression Omnibus (GEO). We applied CIBERSORT and ESTIMATE algorithms to explore the immune landscape. Experiments were performed both in vivo and vitro in using the RM1 murine PCa cell line and C57BL/6 mice model, respectively. Additionally, we examined clinical specimens from Beijing Shijitan Hospital using immunohistochemistry. Figure 1 displayed a flow chart outlining the experimental steps.

### Data processing

In this study, eligible datasets were screened according to the following criteria. Inclusion criteria: (1) The pathological diagnosis was PCa; (2) Availability of raw or normalized transcriptomic data (RNA-seq or microarray); (3) Availability of detailed clinical annotations, including Gleason Score, T stage, and follow-up information for either Biochemical Recurrence-Free Survival (BCRFS) or Distant Metastasis-Free Survival (DMFS). Exclusion criteria: (1) The sample size in small data sets (with a sample size less than 200); (2) Data sets with incomplete or lacking clinical information; (3) Studies consisting only of cell lines or xenograft models.

Three datasets, namely TCGA, GSE116918, and MSKCC, were selected based on specific inclusion and exclusion criteria. Gene expression and clinicopathological data for 961 prostate cancer samples and 52 normal samples were obtained. RNA-seq data and clinicopathological information from TCGA, comprising 495 prostate cancer and 52 paired adjacent normal samples, were retrieved from its portal to serve as the training cohort. The validation cohort consisted of the GSE116918 dataset, which included 248 prostate cancer samples. The test cohort comprised the MSKCC dataset, containing 218 prostate cancer samples. The “Normalize Between Array” function from the R package “limma” was utilized to normalize the datasets. In Table S1, the clinicopathological attributes of the datasets were outlined.

### Screening of the core gene

Cox regression analysis was conducted utilizing the “survival” package on the TCGA and GSE116918 datasets to identify independent prognostic genes associated with BCRFS and DMFS in PCa. Genes with a Benjamini-Hochberg adjusted p-value < 0.05 were considered significantly associated with prognosis. Core genes were determined by intersecting these results with the top 20 key genes identified through the Clustering Coefficient Ranking method in our prior study [18]. The Tumor Immune Estimation Resource (TIMER) was used to assess the expression levels of the core genes in different cancer tissues.

### Prognostic analysis of SLAMF8 in PCa

The prognostic implications of SLAMF8 in PCa were examined through multivariate, univariate Cox regression and survival analyses using the R software packages ‘survminer’ and ‘survival’. We excluded the normal control population, as well as patients with incomplete survival information. 431 tumor samples from 495PCa cases were screened for biochemical recurrence survival analysis and 461 PCa samples for distant metastasis survival analysis in the TCGA database. Biochemical recurrence and distant metastasis survival analyses were conducted on 248 samples from the GSE11691 cohort. Furthermore, 140 samples from MSKCC cohort samples were selected for distant metastasis survival analysis. The Kaplan-Meier technique was employed to draw survival curves. Additionally, clinical information from all three groups of data was combined to study SLAMF8’s clinical implications in prostate cancer.

### Immunological correlation analysis of SLAMF8

This investigation utilized the ESTIMATE method to assess stromal cell content, tumor purity, and immune score in PCa tissue [20]. Additionally, single-sample gene set enrichment analysis (ssGSEA) [21] was applied to quantitatively analyze 29 sets of genes related to immunity [22] in the TCGA cohort.

CIBERSORT algorithm was a method for analyzing the immune cell proportion by using bulk tissue gene expression matrices [23] and served to calculate the percentages of LM22 human immune cell subtypes. The CIBERSORT algorithm was employed to calculate immune cell infiltration levels (CIBERSORT R script v1.03; http://cibersort.stanford.edu/).

Time2.0 is a detailed platform for the automated examination and visualization of immune infiltration levels (https://cistrome.shinyapps.io/timer/) [24]. TIMER algorithm was used to analyze the correlation between SLAMF8, the tumor purity and the expression of six immune cells in PCa. The corrplot package was used to visualize the Spearman correlation analysis results between SLAMF8 and parameters such as tumor purity and immune score.

### GSEA enrichment analysis

Prostate cancer samples from the TCGA were separated into two categories based on the levels of SLAMF8 expression. The analysis was conducted using GSEA software version 4.1.0. to identify the signaling pathways significantly associated with SLAMF8 expression levels [25]. All genes were sorted by their differential expression between the two groups. GSEA evaluates expression profiles across the genome, rather than focusing only on a few predominantly altered genes. The Molecular Signatures Databases were used with GSEA software to analyze KEGG pathways, biological processes (BP), cellular components (CC) and molecular functions (MF). A p value less than 0.05 was regarded as significant for enrichment.

### Immunohistochemistry (IHC) experiments

IHC experiments were approved by the Ethics Committee of Beijing Shijitan Hospital, Capital Medical University (Approval No. IIT2024-091-002). Paraffin-embedded sections were initially subjected to deparaffinization, followed by heating in a microwave oven with 10% citrate buffer for two intervals of 10 min each, after which they were allowed to cool to room temperature. Subsequently, sections were blocked using 5% BSA for a duration of 30 min at room temperature. At 4˚C, sections were incubated overnight with either rabbit anti-SLAMF8 polyclonal antibody (Invitrogen, USA), or mouse polyclonal antibodies targeting PD1, CD3, and CD19 (Zhongshan Golden Bridge, Beijing, China). Following three washes with TBST, the sections were treated with secondary antibodies (Zhongshan Golden Bridge, Beijing, China). After an additional three washes with TBST, the signals were detected using a DAB substrate kit in accordance with the manufacturer’s instructions. A microscope (DM2000; Leica Microsystems GmbH) was used to capture images.Staining intensity was classified into these categories: 0 for negative, 1 for weak, 2 for moderate, and 3 for strong. Additionally, A scale from 0 to 100% was used to quantify the percentage of SLAMF8-positive cells that displayed staining. The H-score, which can range from 0 to 300, was determined by multiplying the staining intensity by the percentage of SLAMF8-positive cells.

### Cell resuscitation and culture

Murine prostate cancer cell line RM1 cells (purchased from Beina Chuanglian Biotechnology Co., LTD., Beijing, China) were taken out of liquid nitrogen and immersed in a 37 °C water bath. The freezing solution was dissolved by gently shaking the freezing tube. After dissolving, the RM1 cell suspension was blended with 1640 medium, which had 10% fetal bovine serum and 1% penicillin-streptomycin, then spun at a speed of 1000 revolutions per minute (rpm) for a duration of 5 min at ambient temperature. The liquid above the cells was discarded, and the cell pellet was collected. Afterward, a new medium was introduced and incubated at 37 °C with 5% CO_2_ in a humid environment.

### Lentiviral synthesis and vector construction

Transcript information for SLAMF8 was selected according to NCBI, and could be found in Table S2 of the supporting information. The target gene vector phBLV-CMV-MCS-3FLAG-EF1-ZsGreen-TDA-puro was constructed by Hanheng Biotechnology Co., and the vector plasmid carrying the target gene and the plasmid were transferred into 293T cells together. The HBLV-m-Slamf8-3xflag-ZsGreen-PURO virus with high expression of SLAMF8 and the control HBLV-ZsGreen-PURO virus were obtained.

### Stably transfected cell lines construction

Cells of the RM1 line, in their logarithmic growth phase and under favorable conditions, were placed into 6-well plates at 5 × 10^4^ cells per well and cultured overnight at 37℃. The RM1 cells were cultured to about 80% confluence. After 48 h, fresh medium was replaced, and then medium containing puromycin (2 µg/ml) was added. The medium with puromycin was refreshed every 2–3 days to replace the one with many dead cells until the resistant cells were selected and cultured. Identification and cryopreservation. The passaged cells were collected, and the mRNA level of SLAMF8 in control RM1 stable cells and RM1 stable overexpressing cell lines was detected by reverse transcription-polymerase chain reaction (RT-PCR).

### Cell viability assay

Overexpression-SLAMF8-RM1 cells (OE-SLAMF8-RM1), control plasmid RM1 cells (CP-RM1) and negative control-RM1 cells (NC -RM1) were seeded at 3 × 10^3^ / well in 96-well cell culture plate and cultured for Two days at 37℃ in an incubator with 5% CO_2_. Then, 10ul of CCK8 reaction solution (APExBIO, USA) was introduced into each well and kept within an incubator set at 37 °C and 5% CO_2_ for a duration of 60 min. Ultimately, the 96-well plate was placed in the microplate reader (BIOBASE) to measure absorbance at 450 nm.

### Cell invasion experiment

The Transwell chamber serves as a crucial tool for assessing the invasion and migration capabilities of cancerous cells. For chamber preparation, 100 µl of Matrigel with a final concentration of 0.5 mg/ml was gently placed in the center of the bottom surface of the upper chamber and incubated at 37 °C until it formed a gel. OE-SLAMF8-RM1, CP-RM1, and NC-RM1 cell suspensions were prepared at a density of 3 × 10^5^ cells/ml. Afterward, 200 µl of each cell suspension was placed into the upper chamber of the Transwell system and incubated at 37 °C with 5% CO_2_. Following a 48-hour incubation, the cells were gently rinsed with phosphate-buffered saline (PBS) after removing the Transwell chamber. Afterward, the cells were fixed for an hour using a 70% solution of ethanol that was kept cold with ice. Post-fixation, a 0.5% crystal violet staining solution was applied for 20 min at room temperature. Following PBS washing, the cells were visualized and quantified under a microscope (Nikon) at 200× magnification.

### Cell cycle and apoptosis experiment

To assess apoptosis, the Annexin V-APC/7-AAD detection kit (Nanjing KGI Biotech Co., LTD.)was utilized. Briefly, OE-SLAMF8-RM1, CP-RM1, and NC-RM1 cells, maintained in optimal growth conditions, were seeded into 6-well plates at a density of 3 × 10^5^ cells per well and incubated for 48 h at 37 °C in a 5% CO_2_ atmosphere. Following trypsinization, the cells were centrifuged at 3000 rpm for 5 minutes. After two washes with PBS, 500 µl of Binding Buffer was added, and the cells were resuspended. Subsequently, 5 µl of Annexin V-APC and 5 µl of 7-AAD were added, and the mixture was incubated at room temperature in the dark for 15 min before analysis by flow cytometry (CytoFLEX, BECKMAN). Similarly, cells were cultured and harvested under identical conditions, washed twice with PBS, fixed with pre-cooled ethanol for 40 min at 4 °C, washed and centrifuged with PBS, treated with RNase (1 mg/ml) and PI (400 µg/ml), and stained for 30 min at 4 °C without light. Ultimately, flow cytometry was used to assess changes in the cell cycle.

### Tumor transplantation model in vivo

Animal experiments, all husbandry and euthanization were approved by the Ethics Committee of Beijing Shijitan Hospital (Approval No. KYD-2024-0070-001). The one-tailed t test required a sample size of 10, assuming α = 0.05, β = 0.35, and an effect size of 0.95 (GPower 3.1.9.7) [26]. All experiments were conducted following the ethical standards and the Declaration of Helsinki. Randomization was carried out using a sequence generated by a computer. The person conducting the experiment did not know the group assignments, and only the main researcher was informed about the group allocations at various phases of the experiment. To reduce possible confounding factors, mice were paired based on age and body weight. Conducting the operation under local anesthesia can substantially reduce animal suffering. The OE-SLAMF8-RM1 cell line and the control cell line were injected subcutaneously into the right axilla of each mice group at a dose of 5 × 10^6^/200ul. Using a vernier caliper, the long (a) and short (b) diameters of the tumor were recorded every three days, and its volume was calculated with formula 1. Simultaneously, the weight of the mouse was recorded every three days. After 21 days, the mouse were euthanized, and their tumor tissues were extracted for photography and weighing.

1 $$\:\text{v}=\frac{\text{a}{\text{b}}^{2}}{2}$$

### Immunohistochemistry (IHC) experiments

Both clinical and mouse tumor tissues were collected and treated with 4% paraformaldehyde for preservation. The tissues underwent dehydration, clearing, infiltration with wax, and embedding processes. Tumor wax blocks were sectioned into 5 μm slices, which were subsequently deparaffinized using xylene for 30 min and rehydrated with ethanol in 5-minute intervals over a 15-minute period. All sections underwent antigen retrieval by being immersed in a sodium citrate solution at about 95 °C for 10 min. Subsequently, a 3% hydrogen peroxide solution was used for 30 min to inhibit endogenous peroxidase activity and minimize background staining. The sections were treated with 5% goat serum for blocking, followed by an overnight incubation at 4 °C with primary antibodies targeting MMP9 and SLAMF8, each diluted to 1:500. PBS was used to wash the sections, which were then incubated with secondary antibodies for 2 h at 4 °C, followed by a 15-minute incubation with 3,3’-diaminobenzidine (DAB) chromogen solution for visualization. Each sample was examined and photographed using an Olympus BX51 microscope (Tokyo, Japan), and the expression levels of MMP9 and SLAMF8 were quantified using ImageJ software.

### Serum inflammatory factors were detected by ELISA

Serum was collected by centrifuging mouse blood at 1500 rpm for 15 min at 4 °C. Serum inflammatory factors, TNF-α and IL-6, were quantified with an ELISA kit (Ruixin Biotechnology Co., LTD., China) in accordance with the manufacturer’s instructions.

### Immune cell detection

Peripheral blood was collected for the preparation of peripheral blood mononuclear cells (PBMCs) using a lymphocyte separation solution (Tianjin Haoyang Biological). The PBMCs were diluted to a concentration of 1 × 10^6^ cells/mL using FACS buffer (PBS containing 0.5% FBS). Subsequently, Antibodies against mouse CD3e, CD4, and CD8a, conjugated with FITC, APC, and PE respectively (eBioscience), were used for surface staining. Staining was executed at 4 °C for a duration of 30 min in the dark, maintaining a speed of 1500 rpm. The cells were centrifuged for 5 min, washed twice with PBS, and then resuspended in 0.2 mL of PBS containing 0.5% BSA. Flow cytometry was then performed for detection and further data analysis.

### RT-PCR detection

An adequate portion of mouse tumor tissue was taken, and Trizol reagent (Biyuntian, Beijing) facilitated the extraction of total RNA. A NanoDrop™2000c spectrophotometer was used to evaluate the RNA’s concentration and purity. The HiScript III 1st Strand cDNA Synthesis Kit (Nanjing Norvelzen Biotechnology Co., LTD.) was used to convert total RNA into cDNA. according to the product instructions. Then, the expression of relevant genes was quantified by adding SYBR Green dye using a Real-Time PCR detection system (ABI 7500). The primer used in the reaction system was synthesized by Shanghai Sangon, and its primer sequence is as follows:

SLAMF8 Forward: 5’-AGACCCAACCCCTCAATACC-3’; Reverse: 5’-GAAGTCCACTGTCCCCTCCC-3’. Actin Forward: 5’-AGAGGGAAATCGTGCGTGA-3’; Reverse: 5’-CATTGCCGATAGTGATGACCT-3’. Data quantification was performed using the 2 − ΔΔCT method, and normalization was done using the β-actin gene as a reference.

### Western blot analysis

Tumor tissues were cut and ground, and RIPA lysate (containing 0.1% PMSF) was added for total protein extraction. The BCA Protein Assay Kit (Biyuntian Bio, China), was used to measure protein concentrations in the samples. After combining the extracted protein supernatant with a 5× protein loading buffer in a 4:1 volume ratio, it was denatured by boiling for 10 min. Electrophoresis was performed on a 12% SDS-PAGE gel using 20 µg of protein from each sample, followed by transfer onto a PVDF membrane using a transmembrane apparatus. In a sealed container, the PVDF membrane was blocked with TBST that included 5% skim milk powder for 2 h at room temperature. The membranes were then incubated overnight at 4 °C with primary antibodies: rabbit anti-mouse TLR4 (1:1000), rabbit anti-mouse NF-κB p65 (1:2000), and rabbit anti-mouse GAPDH. Following incubation, the PVDF membrane was subjected to five 5-minute washes with TBST, and then incubated with a secondary antibody, goat anti-mouse IgG-HRP (1:10000), diluted in TBST, for 2 h at room temperature on a shaker. Five thorough washes of the membrane were performed using TBST, each lasting 5 min. Finally, protein bands were visualized using an ECL chemiluminescence detection kit (Thermo Fisher Scientific, USA) and then exposed on X-ray film.

### Targeted therapy drug sensitivity analysis

The association between SLAMF8 expression levels and the 50% inhibitory concentration (IC50) of targeted therapeutic agents was evaluated utilizing the “pRRophetic” R package (version 0.5, available at https://github.com/paulgeeleher/pRRophetic, accessed on July 6, 2024). IC50 data were sourced from the study conducted by Garnett et al. [27]. The Wilcoxon test was applied to compare sensitivity differences between PCa patients exhibiting high versus low SLAMF8 expression, facilitating the identification of targeted therapeutic agents significantly correlated with SLAMF8 expression (*p* < 0.001). The TCIA database provided the immunotherapy score data.

### Statistics

Data were analyzed using R version 3.6.3. The relationship between SLAMF8 expression and clinical variables was examined using Chi-square or Fisher’s least significant difference (LSD) exact tests. A Student’s t-test assessed the normal distribution of continuous indicators between two groups, while a non-parametric test checked partial distributions. One-way analysis of variance (ANOVA) compared multiple groups. Pearson’s correlation measured the link between SLAMF8 expression and clinical variables. Univariate and multivariate Cox regression models evaluated SLAMF8 as an independent prognostic indicator in PCa patients. Kaplan-Meier curves assessed SLAMF8’s prognostic significance, and time-dependent receiver operating characteristic (TD-ROC) analysis evaluated its predictive ability for BCRFS and DMFS. Statistical significance was shown by a two-sided P value below 0.05.

### Data availability

The raw transcriptome and clinical data have been deposited in the TCGA and the GEO program. The corresponding authors will share all necessary data upon a reasonable request.

## Results

### Screening procedure of SLAMF8 and its expression pattern in PCa

**Fig. 2:**
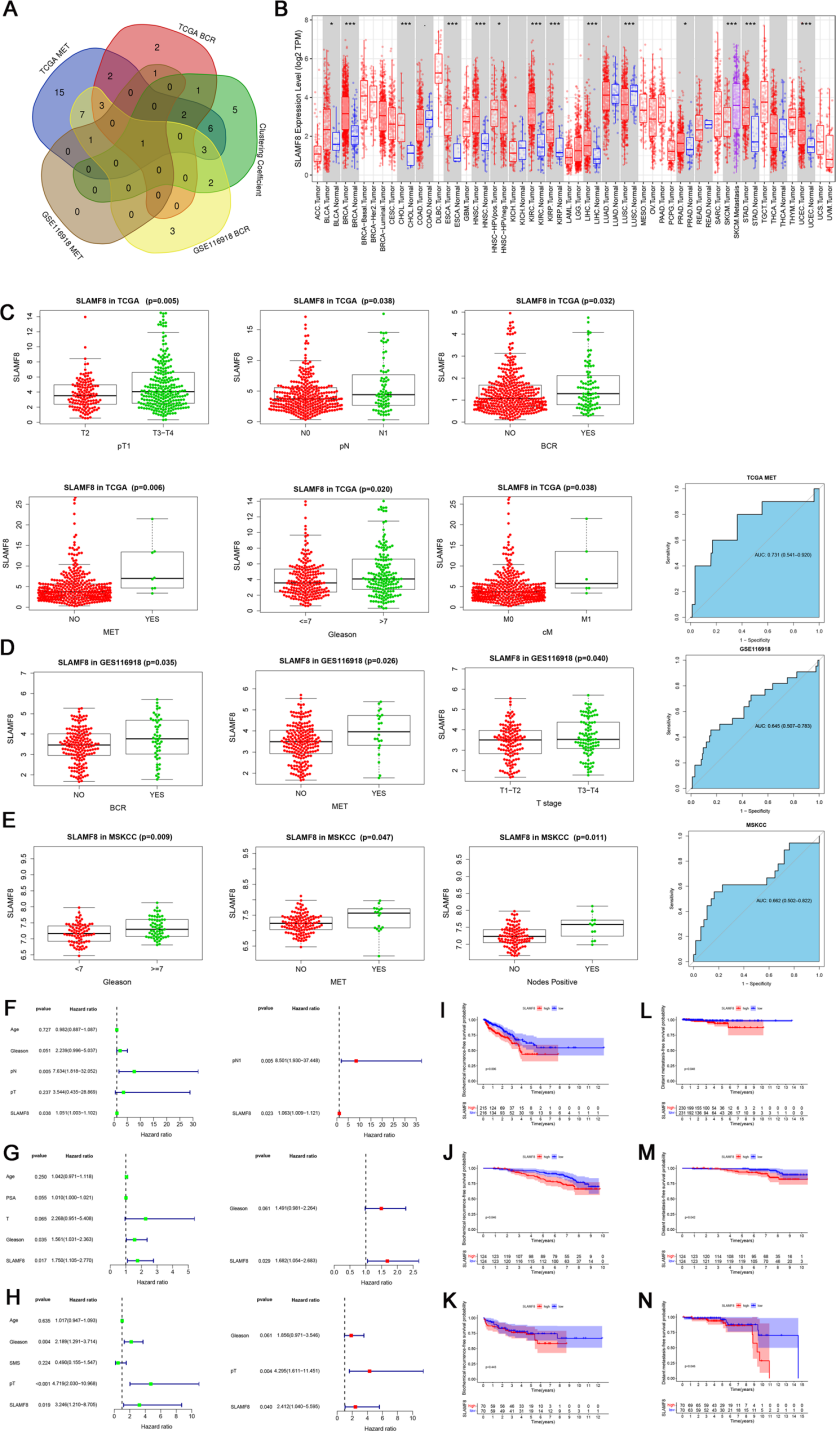
Clinical correlation and prognostic analysis of SLAMF8. (**A**) Univariate Cox analysis on the TCGA and GSE116918 datasets was performed to discover independent prognostic genes associated with biochemical recurrence and distant metastasis, and a Venn diagram was utilized to intersect these with 20 key genes; (**B**) TIMER analysis of SLAMF8. The comparison of gene expression levels is shown by boxplot. SLAMF8 is highly expressed in Prostate adenocarcinoma (PRAD), Bladder urothelial carcinoma (BLCA) and other cancers; (**C**) increased with the malignant degree of SLAMF8 expression quantity also increased significantly, as markers in the diagnosis of prostate cancer metastasis AUC is 0.731; (**D**) SLAMF8 expression in GSE116918 dataset showed that SLAMF8 expression rose notably as the malignancy level increased, and the AUC of SLAMF8 as a marker for the diagnosis of prostate cancer metastasis was 0.645. (**E**) The clinical expression analysis of SLAMF8 in the MSKCC dataset showed that an increase in the malignancy degree led to a marked rise in SLAMF8 expression, and the AUC of SLAMF8 as a marker for the diagnosis of prostate cancer metastasis was 0.662. MET, Metastasis. (**F**) In TCGA dataset, pN stage and SLAMF8 were identified as independent prognostic factors through Cox regression analyses; (**G**) In GSE116918 dataset, Cox regression analysis found SLAMF8 was an independent prognostic factor, excluding Gleason; (**H**) In MSKCC dataset, the Cox analysis demonstrated that pT stage and SLAMF8 were independent prognostic factors, but not Gleason; (**I**-**J**) TCGA and GSE116918 datasets, SLAMF8 was significantly associated with BCRFS, (**K**) In the MSKCC dataset, SLAMF8 expression was not significantly correlated with BCRFS. (**L**-**N**) In all three datasets, a significant reduction in metastasis-free survival was associated with the overexpression of SLAMF8

Univariate Cox regression was applied to the TCGA dataset to find independent prognostic genes linked to biochemical recurrence and distant metastasis. We identified 12 genes associated with biochemical recurrence (Table S3) and 40 genes related to distant metastasis (Table S4). Then, in the GSE116918 data set, 20 genes associated with biochemical recurrence were found through univariate Cox analysis (Table S5) and 3 genes were related to distant metastasis (Table S6). These four gene sets intersected with the 20 key genes from our previous studies (Table S7). The Venn diagram was obtained with the core gene SLAMF8 (Fig. 2A). According to the TIMER database’s pan-cancer differential gene expression analysis, SLAMF8 was later found to be highly expressed in prostate cancer and various other cancers, as shown in Fig. 2B. To investigate the link between SLAMF8 expression and prostate cancer clinical characteristics, researchers categorized individuals into high and low groups using the median cut method. The relationship between SLAMF8 expression and clinical characteristics of PCa patients was assessed using a chi-square test, which demonstrated a strong correlation. GEPIA2 analysis of TCGA RNA sequencing data in 495 cancer tissues and 52 normal tissues was carried out in TCGA. The expression of SLAMF8 was up-regulated in groups with higher N stage (*P* = 0. 038), T stage (*P* < 0.005), BCR (*P* = 0. 032) and Gleason score (*P* = 0.020). Expression levels were greater in the metastatic group than in the non-metastatic group (*P* = 0.006). SLAMF8 levels are useful for metastatic prostate cancer (AUC = 0.731) (Fig. 2C). In the GSE11691 dataset, a nonparametric test analysis revealed that SLAMF8 levels were markedly higher in the T3-4 group than in the T1-2 group (*P* = 0.040). SLAMF8 expression was notably higher in the metastatic group (*P* = 0.026) and the BCR group (*P* = 0.035). Metastatic prostate cancer (AUC value = 0.645) can be diagnosed using SLAMF8 levels (Fig. 2D). Meanwhile, we also found that SLAMF8 expression was elevated in cases with higher Gleason scores (*P* = 0.009), distant metastasis (*P* = 0.047), and lymph node metastasis (*P* = 0.011) (Fig. 2E). There was an AUC value of 0.662 for metastatic prostate cancer in MSKCC cohort. Briefly, the expression of SLAMF8 increased as PCa malignant degree increased. We assessed SLAMF8’s prognostic value in prostate cancer across TCGA (*n* = 495), GSE11691 (*n* = 248), and MSKCC (*n* = 140) cohorts. Our findings indicate that SLAMF8 is an independent prognostic factor for distant metastasis in PCa, as shown by in the TCGA cohort (HR = 1.063, *P* = 0.023) (Fig. 2F), GSE11691 cohort (HR = 1.682, *P* = 0.029) (Fig. 2G), and MSKCC cohort (HR = 2.412, *P* = 0.040) (Fig. 2H). COX regression analysis confirmed SLAMF8’s significant association with metastasis-free survival, and survival analysis linked its expression to poor DMFS across all three cohorts (Fig. 2I-N). Consequently, SLAMF8 functions as a prognostic predictor for those with prostate cancer. While SLAMF8 demonstrated significant prognostic value for metastasis-free survival across all cohorts (HR > 1.06, *P* < 0.05), its diagnostic accuracy for existing metastasis was modest (AUC = 0.645–0.731). This underscores its primary utility as a prognostic—rather than diagnostic—biomarker, identifying patients at risk of future metastatic progression.

### Relationship between SLAMF8 and immunity

**Fig. 3:**
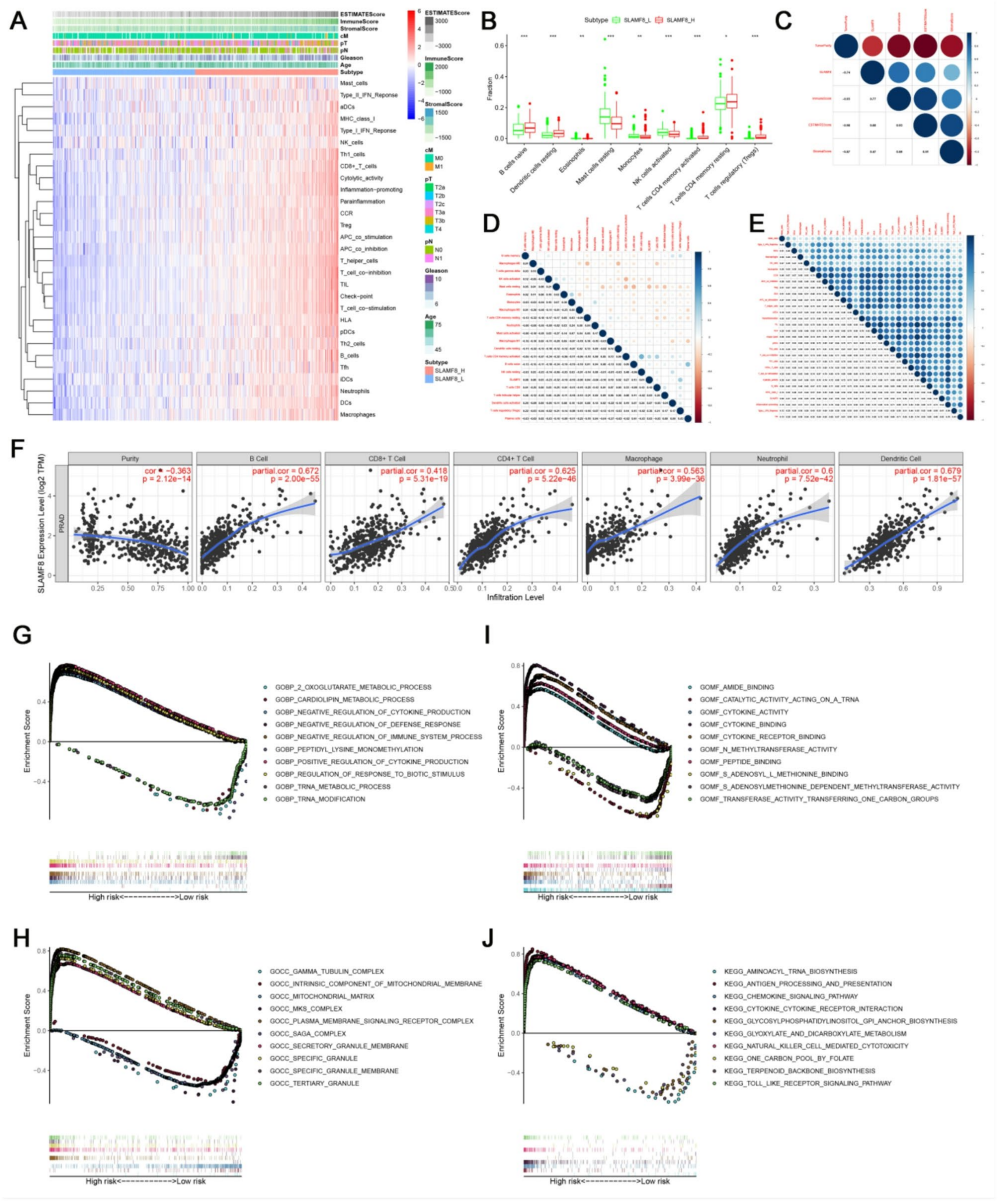
Relationship between immune cell infiltration and SLAMF8. (**A**) The ‘ESTIMATE’ analysis provided a molecular cluster map of SLAMF8 expression involving 29 different immune cells; (**B**) Box plot of SLAMF8 immune cell infiltration obtained by “CIBERSORT” analysis. Nine immune cell types were significantly correlated with the expression of SLAMF8; (**C**) Assessment of the correlation between SLAMF8 expression and immune score, tumor purity, ESTIMATE and stromal score; (**D**) Analysis of the correlation between immune cell and SLAMF8 expression; (**E**) Study of how SLAMF8 expression correlates with immune checkpoints; (**F**) The TIMER database facilitated the assessment of the link between SLAMF8 expression, tumor purity, and immune cell infiltration. (**G**) DEGs highlight the top five enriched BP; (**H**) Top five CC enriched by DEGs; (**I**) The top five MF enriched by DEGs; (**J**) Top five KEGG pathways by DEGs

The molecular landscape of SLAMF8 expression with 29 immune cells was obtained by ESTIMATE analysis (Fig. 3A): In the SLAMF8 group with low expression, the expression of immune cells also decreased. SLAMF8 is highly expressed in low tumor purity group. According to the findings, SLAMF8 could potentially predict the immune status of prostate cancer. Utilizing the CIBERSORT algorithm, SLAMF8 expression showed a strong relationship with various immune cell types (Fig. 3B). Pearson’s correlation analysis further substantiated that SLAMF8 expression is intricately linked to immune score, tumor purity, ESTIMATE score, stromal score, immune cell expression, and immune checkpoint activity. Notably, SLAMF8 was closely linked to the expression of different immune cells, with correlation coefficients exceeding 0.7. This includes T helper cells (*r* = 0.7), tumor-infiltrating lymphocytes (TILs) (*r* = 0.71), immune checkpoints (*r* = 0.71), T cell co-stimulation (*r* = 0.71) and T cell co-inhibition (*r* = 0.73) (Fig. 3C-E). Furthermore, In Fig. 3F, the TIMER database is used to evaluate SLAMF8 mRNA expression and immune cell infiltration. SLAMF8 levels in peripheral blood were negatively correlated with tumor purity (*r*=-0.363, *P* = 2.12 × 10^14^) and positively correlated with B cells (*r* = 0.672, *P* = 2.00 × 10^55^), CD8 + T cells (*r* = 0.418, *P* = 5.31 × 10^19^), CD4 + T cells (*r* = 0.625, *P* = 5.22 × 10^46^), macrophages (*r* = 0.563, *P* = 3.99 × 10^36^), neutrophils (*r* = 0.60, *P* = 7.52 × 10^42^), and dendritic cells (*r* = 0.679, *P* = 1.81 × 10^57^). The above findings suggest that SLAMF8 is closely related to T and B cell immunity. The proliferation, differentiation, activation, and receptor signaling pathways of T cells are positively correlated with SLAMF8. Meanwhile, We also found a positive association between: SLAMF8 and B cell activation. SLAMF8 is important for the regulation of T cell immunity and also affects immune cells (dendritic cells and neutrophils, macrophages).

Differentially expressed genes (DEGs) in groups with high and low SLAMF8 expression were analyzed using GSEA enrichment. We explore the biological processes that might be associated with SLAMF8 mRNA enrichment. For biological processes (BP), high-SLAMF8 DEmRNA group were mainly focused on positive regulation of cytokine production, regulation of response to biological stimuli, and negative regulation of immune system processes (Fig. 3G). For cellular components (CC), these DEmRNAs were significantly correlated mainly by special granules, tertiary granules, special granule membranes (Fig. 3H). Furthermore, these are the major biochemical functions of DEmRNA (MF): peptide binding, cytokine receptor binding, cytokine activity (Fig. 3I). Based on the KEGG analysis, there are three major pathways involved in natural killer cell mediated cytotoxicity, antigen processing and presentation, Toll-like receptor signaling pathway (Fig. 3J).

### Results of immunohistochemistry

**Fig. 4:**
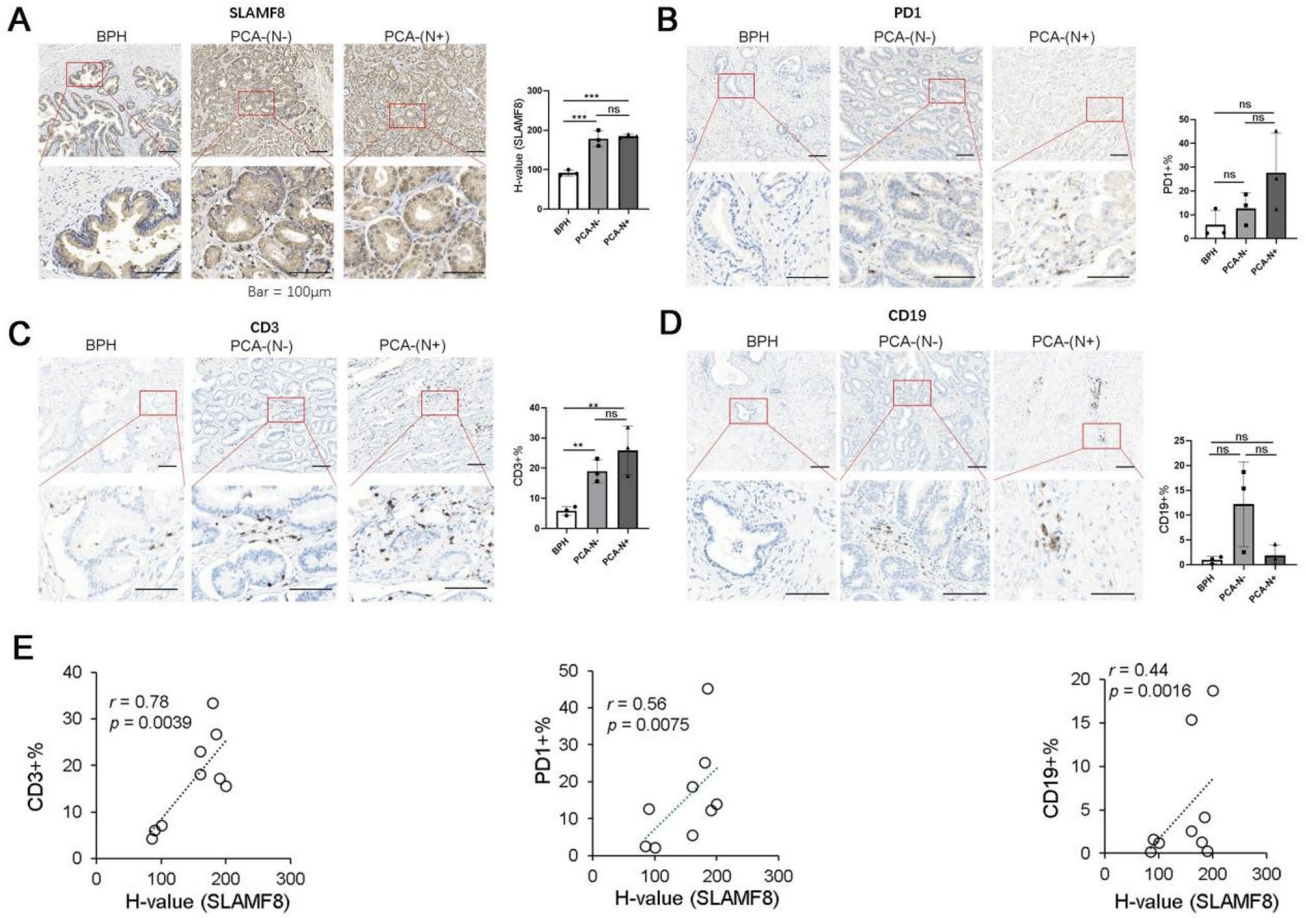
Immunohistochemical results of SLAMF8 and immune cells in prostate tissue. (**A**) The level of SLAMF8 in PCa and BPH tissues were examined by immunohistochemistry. Images were obtained using a microscope. The evaluation was performed separately for staining intensity, with 0 as negative, 1 as weak, 2 as moderate, and 3 as strong, and for the percentage of SLAMF8 positive cells that demonstrated staining (0 to 100%). An H-score ranging from 0 to 300 was calculated by multiplying the positivity intensity by the percentage of SLAMF8 positive cells that exhibited staining. The H-score were calculated by using 5 independent images per sample. Bar = 100 μm; (**B**) An antibody targeting PD1 was employed in immunohistochemistry to evaluate the number of PD1 positive cells in PCa and BPH tissues. Using a microscope, images were acquired. The percentages of T cells were calculated by using 5 independent images per sample. Bar = 100 μm; (**C**) The number of T cells in PCA and BPH tissues were examined by immunohistochemistry by using antibody targeting CD3. Images were obtained using a microscope. The percentages of T cells were calculated by using 5 independent images per sample. Bar = 100 μm. (**D**) The number of B cells in PCA and BPH tissues were examined by immunohistochemistry by using antibody targeting CD19. Images were obtained using a microscope. The percentages of T cells were calculated by using 5 independent images per sample. Bar = 100 μm. (**E**) SLAMF8 expression level and CD3, PD1, CD19% of positive cells were positively correlated

To assess SLAMF8 levels in PCa and examine its association with immune cell infiltration, we employed immunohistochemistry to measure the levels of SLAMF8, PD1, CD3, and CD19 in both PCa and benign prostatic hyperplasia (BPH) samples. Our study found a marked escalation in: SLAMF8 expression and the percentage of CD3-positive cells in PCa samples. Furthermore, SLAMF8 expression demonstrateda proportional connection with the percentages of CD3-positive, PD1-positive, and CD19-positive cells. However, the numbers of PD1-positive and CD19-positive cells were not significantly elevated in PCa tissues. The findings indicate that increased SLAMF8 expression in PCa tissues boosts the infiltration of B cells and T cells (Fig. 4).

### SLAMF8 enhances cell proliferation and movement while reducing apoptosis in prostate cancer cells through the activation of the NF-κB signaling pathway

**Fig. 5:**
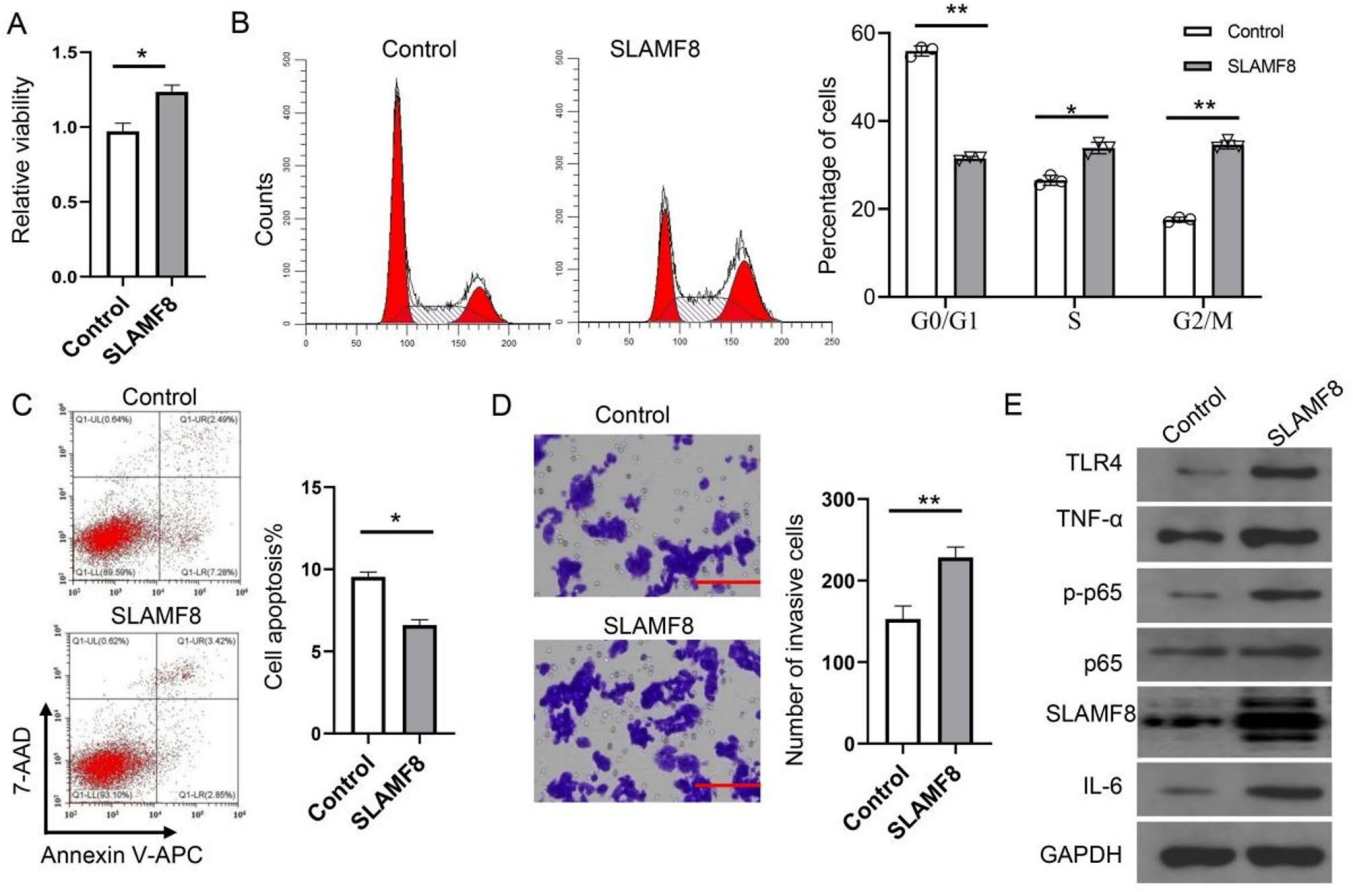
SLAMF8 promotes PRAD cells proliferation via activating NF-κB signaling. (**A**) CCK8 assay to detect cell viability; (**B**) Cell cycle analysis; (**C**) Flow cytometry was used to identify apoptotic cells following labeling with 7-AAD and Annexin-V; (**D**) Transwell assay to detect cell migration capacity; (**E**) Immunoblotting analysis

SLAMF8 overexpressed cells showed significantly higher cell viability than control cells Fig. 5A. More of the SLAMF8 overexpressed cells distributed into S and G2/M phases of cell cycle (Fig. 5B). SLAMF8 overexpression inhibited the percentage of apoptotic cells (Fig. 5C). Furthermore, the migration capacity of SLAMF8 overexpressed cells was significantly enhanced by overexpression of SLAMF8 (Fig. 5D). Consistent with activation of the NF-κB signaling pathway, SLAMF8 overexpressing cells demonstrated upregulated TLR4 expression, increased phosphorylation of p65, and elevated secretion of IL-6 and TNF-α (Fig. 5E). While these findings establish a functional relationship between SLAMF8 and NF-κB pathway activation, the precise molecular intermediates require further investigation.

### SLAMF8 contributes to the expansion of tumors in vivo

**Fig. 6:**
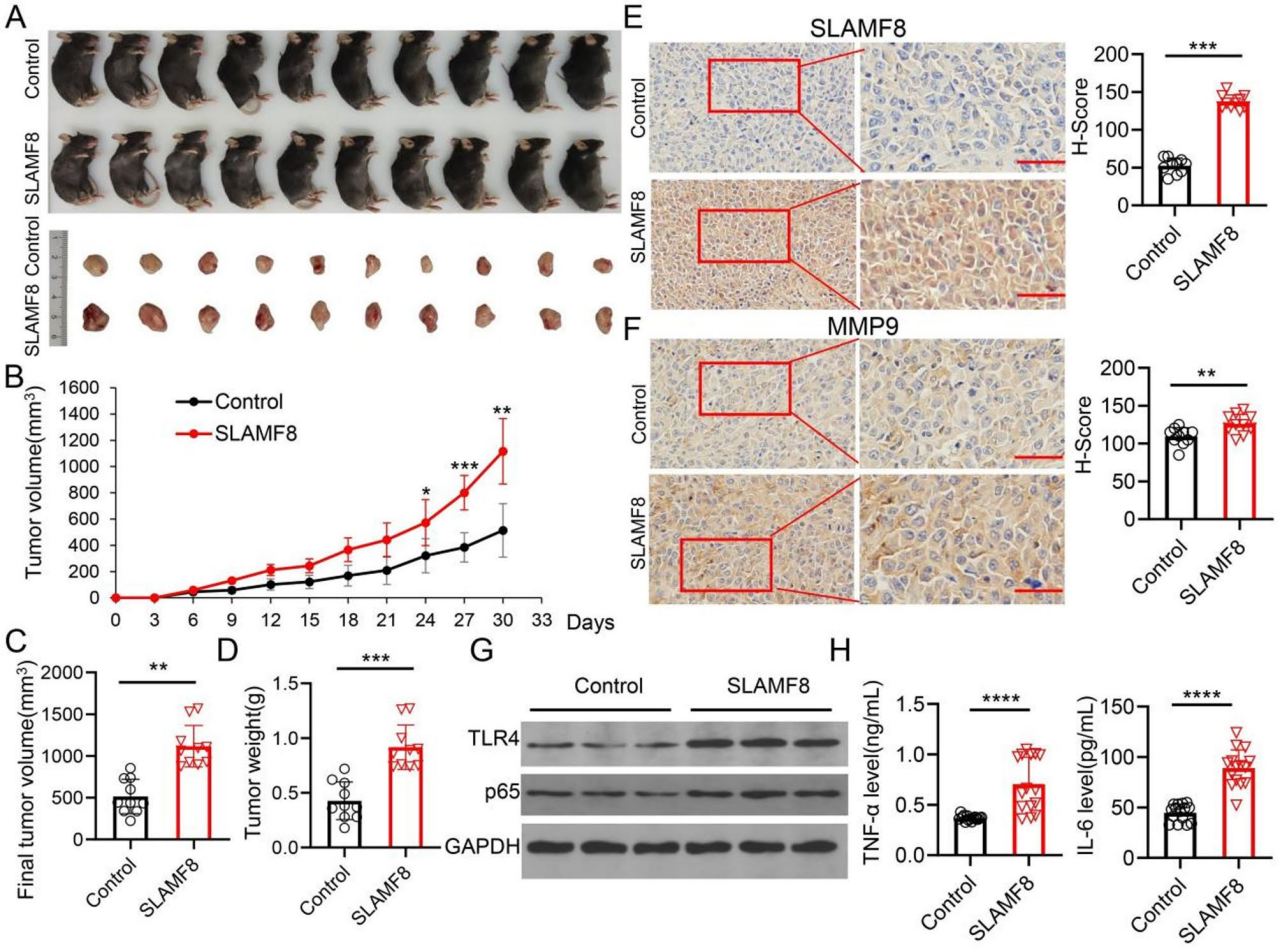
SLAMF8 promotes PRAD growth in vivo. (**A**) Tumors from C57BL6 mice and their respective groups were illustrated; (**B**) Curves were used to display tumor growth, and the volume was determined every three days after injection; (**C**) Tumor final volume; (**D**) Tumor weight; (**E**) IHC detecting SLAMF8 level in tumors. Results were quantified and shown as H-score; (**F**) IHC detecting MMP9 level in tumors. Results were quantified and shown as H-score; (**G**) TLR4 and p65 expression in tumors were detected using immunoblotting; (**H**) Serum levels of IL-6 and TNF-α were quantified through ELISA

SLAMF8 overexpressed RM1 cells were injected into male C57/BL6 mice (28–42 days old) subcutaneously (Fig. 6A). The tumor growth was monitored every three days (Fig. 6B). We observed that SLAMF8 overexpression promoted tumor growth significantly (Fig. 6C, D). SLAMF8 overexpression tumors expressed increased MMP9 suggesting a distant metastasis potential (Fig. 6E, F). SLAMF8 overexpression tumors expressed upregulated TLR4 (Fig. 6G) and increased TNF-α and IL-6 (Fig. 6H) indicating activated NF-κB signaling.

### Analysis of drug sensitivity and immunotherapy

**Fig. 7:**
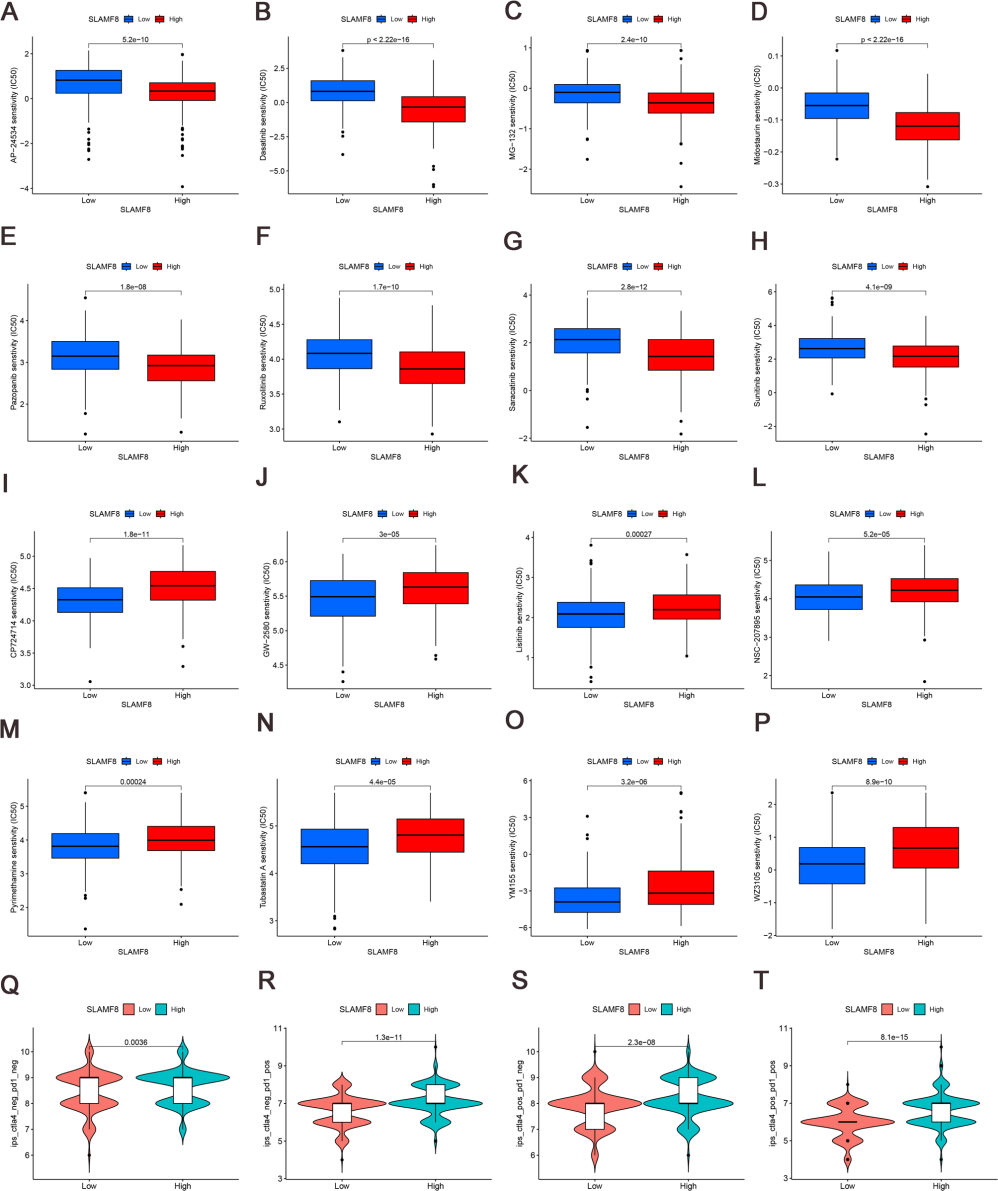
Examining drug sensitivity and immunotherapy in PCa patients based on SLAMF8 expression levels. (**A**–**H**). The IC50 values of drugs sensitive to high SLAMF8 expression are presented in box plots, including AP-24,534 (**A**), Dasatinib (**B**), MG-132 (**C**), Midostaurin (**D**), Pazopanib (**E**), Ruxolitinib (**F**), Saracatinib (**G**), and Sunitinib (**H**); (**I**–**P**). Box plots showing the IC50 values of sensitive drugs in the low SLAMF8 expression group, including CP724714 (**I**), GW-2580 (**J**), Lisitinib (**K**), NSC-207,895 (**L**), Pyrimethamine (**M**), Tubastatin A (**N**), WZ3105 (**O**) and YM155 (**P**); (**Q**–**T**). The immunotherapy scores for PD1, CTLA4 single positive, PD1/CTLA4 double negative or positive patients are higher among those with elevated SLAMF8 expression

We have conducted a prediction of the sensitivity of PCa patients with varying expression levels of SLAMF8 to a range of common anticancer drugs, chemotherapeutic agents, and targeted therapies. Patients exhibiting elevated SLAMF8 expression levels show reduced IC50 values for AP-24,534, Dasatinib, MG-132, Midostaurin, Pazopanib, Ruxolitinib, Saracatinib, and Sunitinib compared to those with low SLAMF8 expression, suggesting a heightened sensitivity to these drugs (Fig. 7A-H). Conversely, the low expression group displays greater sensitivity to Pyrimethamine, CP724714, GW-2580, Lestinib, NSC-207,895, Tubastatin A, WZ3105, and YM155 (Fig. 7I-P). Notably, patients with high SLAMF8 expression who are single-positive for PD1 or CTLA4, or double-negative or double-positive for both markers, exhibit elevated immunotherapy scores, hinting at the potential for immunotherapy to benefit this subgroup of PCa patients (Fig. 7Q-T).

## Discussion

The primary cause of death in prostate cancer patients is the metastasis, and there is a notable deficiency in biomarkers capable of predicting PCa metastasis. Our study analyzed high-throughput sequencing data from the TCGA portal and verified the findings using datasets from the GEO DataSets portal. Our observations revealed that SLAMF8 is significantly overexpressed in PCa tissues, with its expression positively correlating with malignancy and metastatic progression. Low expression of SLAMF8 was found to be significantly connected to a favorable prognosis in prostate cancer patients, as shown by the Cox regression model. Our findings further confirmed that SLAMF8 expression serves as an independent prognostic factor for BCR and DMFS. The degree of immune infiltration and various immune markers exhibited significant correlations with SLAMF8 expression in PCa. Finally, through cell and animal experiments, we further corroborated the association of SLAMF8 with prostate cancer progression and metastasis. From our perspective, our analyses introduce new insights regarding the prognostic significance of SLAMF8 in PCa. Although SLAMF8’s diagnostic accuracy for existing metastasis was suboptimal (AUC < 0.7), its independent prognostic value for MFS (HR = 1.06–2.41; *P* < 0.05) highlights its role in early metastatic programming. This aligns with biological evidence that SLAMF8 promotes pre-metastatic niche formation via TLR4–NF-κB signaling.

SLAMF receptors have emerged as significant players in the landscape of cancer prognostics and therapeutics. These receptors, predominantly expressed on immune cells, are integral to modulating immune responses within the tumor microenvironment, thereby influencing cancer progression and patient outcomes. The SLAMF family consists of nine distinct receptors, each with unique structural and functional characteristics that contribute to their role as potential prognostic markers in various malignancies [28]. For instance, SLAMF7 has been identified as a potential therapeutic target in multiple myeloma, with its expression correlating with disease progression [29]. SLAMF9 has been associated with poor prognosis in colorectal cancer, where its elevated expression correlates with reduced patient survival and increased tumor aggressiveness [30]. The SLAM family, a subset of the broader CD2 protein family, exhibits differential expression on the surface of immune cells [31].SLAMF8, a type I cell surface glycoprotein, has been observed to be overexpressed in conjunction with malignancy progression and serves as a biomarker for the mesenchymal subtype of glioma [17]. It has been observed that SLAMF8 is largely expressed on tumor-associated macrophages in gastrointestinal cancer, contributing to the tumor’s immunosuppressive microenvironment [11]. Nonetheless, SLAMF8’s role in PCa is not yet clearly understood. Our study revealed that SLAMF8 is expressed in PCa tumor cells, with its overexpression supporting tumor expansion. Furthermore, SLAMF8 expression is positively correlated with immune cell infiltration and tumor metastasis in PCa patients. To the best of our knowledge, this is the inaugural report elucidating the role of SLAMF8 in PCa, thereby offering a potential target for PCa treatment.

Chronic activation of Toll-like receptor 4 (TLR4) signaling within the prostate tumor microenvironment is a recognized driver of disease progression, linking innate immune activation to oncogenic processes [32]. A key mechanism involves bacterial lipopolysaccharide (LPS), a canonical TLR4 ligand, which enhances prostate cancer (PCa) cell survival under nutrient-deprived conditions—a common feature of growing tumors [33]. Critically, this LPS/TLR4 axis also induces the overexpression of the chemokine CCL2. This highlights a vital cross-talk between tumor cells and the immune stroma: CCL2 secretion inhibits starvation-induced apoptosis of tumor-associated macrophages (TAMs) and acts as a potent chemoattractant, significantly increasing macrophage infiltration into prostate tumors in vivo [33]. This expanded population of TAMs, often polarized to a pro-tumorigenic (M2) state, further fuels tumor growth, angiogenesis, and immunosuppression, creating a feed-forward loop that accelerates disease progression. The therapeutic relevance of this pathway is underscored by studies showing that genetic knockdown of TLR4 or its downstream effector COX-2 significantly impairs PCa cell proliferation, migration, and invasion by inhibiting the phosphorylation and activation of NF-κB p65 [34, 35]. Furthermore, endogenous ligands like Peroxiredoxin-1 can activate TLR4, stimulating tumor angiogenesis and expansion in murine models, solidifying the role of TLR4-mediated inflammation in PCa pathogenesis [32].

The metastatic program regulated by NF-κB involves the upregulation of key effector genes such as TWIST1 (epithelial-mesenchymal transition), MMP13 (extracellular matrix remodeling), and IL11 (bone metastasis niche formation), with inhibitors like LY2409881 and JSH-23 blocking this pathway [14]. The mechanism of TLR4 activation is initiated when LPS forms a complex with the binding protein CD14. This LPS-CD14 complex then recruits and binds to the TLR4/MD2 receptor complex on the cell surface. This binding induces a critical conformational change in TLR4, facilitating the dimerization and recruitment of intracellular adaptor proteins (MYD88, TRIF), which ultimately triggers downstream NF-κB signal transduction and pro-metastatic gene expression [36]. Our findings position SLAMF8 as a novel upstream regulator of this critical pathway in PCa. We demonstrate that SLAMF8 overexpression in PCa cells leads to increased TLR4 expression, consequent activation of NF-κB signaling, and elevated secretion of pro-inflammatory cytokines like IL-6 and TNF-α. This suggests that SLAMF8 potentiates the TLR4-NF-κB axis, contributing to an immunosuppressive tumor microenvironment and fostering the conditions for metastatic spread, thereby mechanistically linking this immune checkpoint to aggressive disease. Additionally, chronic inflammatory state can create a tumor-promoting microenvironment, and insights from other disease models, such as autoimmune thyroiditis, underscore the profound impact of chronic inflammation on cellular physiology [37].

Low or physiological doses of TLR agonists have been implicated in cancer development. Specifically, in prostate cancer, the expression of TLR4 and its association with chronic inflammation, such as that mediated by interleukin-6 (IL-6). Furthermore, cytokines resulting from TLR signaling are involved in the development and progression of prostate cancer [32]. IL-6, a cytokine with many roles, is typically present in a range of tumor microenvironments and has been associated with tumor development and therapeutic resistance across multiple cancer types, primarily through its modulation of the immune system [38, 39]. Persistent IL-6 activity can promote immune-suppressive signals and hinder the establishment of an effective immune response against cancer cells [40, 41]. SLAMF8 is important for the regulation of T cell immunity and also affects other immune cells (dendritic cells and neutrophils, macrophages). The complex interplay between different immune cell populations and inflammatory mediators is a hallmark of many diseases, with eosinophils and IgE levels, for instance, being key biomarkers in allergic inflammation [42]. Understanding these intricate networks in the TME is crucial for developing effective immunotherapies. In this study, the overexpression of SLAMF8 in prostate cancer cells caused an increase in TLR4 expression, triggered the NF-κB signaling pathway, and led to higher IL-6 secretion, potentially contributing to an immunosuppressive tumor microenvironment. While our study demonstrates that SLAMF8 overexpression activates TLR4-NF-κB signaling functionally, the exact molecular mechanism by which SLAMF8 engages this pathway warrants further investigation. Alternatively, SLAMF8 might regulate TLR4 expression or membrane localization through yet undefined mechanisms. These potential mechanisms represent important directions for future research that would further strengthen the mechanistic claims presented here.

Strategies to enhance or block TLRs could represent promising therapeutic avenues. The discovery of agents that induce selective cytotoxicity in cancer cells, such as plant-derived extracts that activate the P21 pathway, highlights the potential for targeting specific oncogenic mechanisms [43]. This approach aligns with the need for more precise interventions in cancer treatment. Notably, PCa patients expressing SLAMF8 show a good response to immunotherapy, and drugs targeting the NF-κB pathway exhibit lower IC50 values. For instance, the protease inhibitor MG-132 has various functions in the body [44]. MG-132 plays a role in regulating the NF-κB activation pathway [44, 45]. SLAMF8-positive prostate cancer patients may benefit from immunotherapy with MG-132-related drugs, but further studies are necessary to validate this conclusion.

The limitations of this study include the following: 1. While our data nominates SLAMF8 as a compelling contributor to prostate cancer metastasis, its journey to clinical application requires rigorous validation. The immediate next steps include confirming these findings in large-scale, prospectively collected clinical cohorts with well-annotated metastatic outcomes. 2.The development of neutralizing antibodies or small-molecule inhibitors against SLAMF8 would be necessary to preclinically validate its therapeutic tractability in vivo. 3. SLAMF8’s modest diagnostic AUC (0.645–0.731) reflects the inherent limitation of single-gene biomarkers in detecting existing metastasis. Future studies should validate its utility within multi-analyte panels (e.g., combined with PSA, imaging, or circulating tumor DNA) to enhance diagnostic precision. 4. The GSE116918 and MSKCC datasets used in this study do not specify the exact pathological subtypes of prostate cancer (e.g., acinar adenocarcinoma, ductal adenocarcinoma, etc.). While this is a common limitation in large public genomic cohorts, our findings are presented in the context of prostate cancer broadly, and future studies with more detailed pathological annotations may help refine the prognostic and mechanistic role of SLAMF8 across specific subtypes.

## Conclusions

Consequently, we have discovered a new mechanism that involves the SLAMF8-TLR4-NF-κB signaling pathway in the dissemination and invasion of prostate cancer. The target gene, SLAMF8, emerges as a promising candidate for drug targeting and holds substantial potential for informing personalized and precise therapeutic strategies for prostate cancer patients.

## Electronic Supplementary Material

Below is the link to the electronic supplementary material.

**Supplementary Material 1:** Tables S1–S7

## Data Availability

The data and materials used to support the findings of this study are available from the corresponding author upon request.
