# Supplementary material for: Mechanistic insights into SLAMF8-mediated prostate cancer metastasis via the TLR4-NF-κB pathway
Source: J Transl Med. 2025 Oct 29;23:1189. doi: 10.1186/s12967-025-07234-3 (PMC12573839; doi:10.1186/s12967-025-07234-3)
Supplement: Supplementary file 1 — Supplementary Material 1: Tables S1–S7 [file 12967_2025_7234_MOESM1_ESM.docx]

**Table S1 Clinical and pathological characteristics of TCGA and GEO datasets**

| **Group** | **TCGA （n=495）** | **GSE116918 （n=248）** | **MSKCC（n=218）** |
| --- | --- | --- | --- |
| Age | n （%） | n （%） | n （%） |
| ≤60 | 222（44.8） | 35（14.1） | 131（61.1） |
| >60 | 273（55.2） | 213（85.9） | 87（39.9） |
| PSA | n （%） | n （%） | n （%） |
| ≤10 | — | 50（20.2） | 146（67.0） |
| >10 | — | 198（79·8） | 67（30.7） |
| NA |  |  | 5（2.3） |
| T stage | n （%） | n （%） | n （%） |
| T1 | — | 51（20·6） | 104（47.7） |
| T2 | 187（37·8） | 76（30·6） | 88（40.4） |
| T3 | 291（58·8） | 92（37·1） | 16（7.3） |
| T4 | 10（2·0） | 4（1·6） | 1（0.5） |
| NA | 7（1·4） | 25（10·1） | 9（4.1） |
| N stage | n （%） | n （%） | n （%） |
| N0 | 344（69·5） | — | 148 |
| N1 | 78（15·8） | — | 30 |
| NA | 73（14·7） | — | 40 |
| Gleason score | n （%） | n （%） | n （%） |
| <7 | 45（9·1） | 42（17·0） | 106（48.7） |
| =7 | 246（49·7） | 99（39·9） | 77（35.3） |
| >7 | 204（41·2） | 107（43·1） | 34（15.6） |
| NA | — | — | 1（0.4） |
| BCR | n （%） | n （%） | n （%） |
| NO | 397（80.2） | 192（77.4） | 61（28.0） |
| YES | 95（19.2） | 56（22.6） | 137（62.8） |
| NA | 3（0.6） | — | 20（9.2） |
| MET | n （%） | n （%） | n （%） |
| NO | 444（89·7） | 226（91·1） | 181（83.0） |
| YES | 17（3·4） | 22（8·9） | 37（17.0） |
| NA | 34（6·9） | — | — |

**NOTE：PSA, Prostate specific antigen; BCR, Biochemical recurrence; MET,** **metastasis; NA, Not applicable**

**Table S2 Transcript information for SLAMF8**

| Gene Information | Nucleotide sequences |
| --- | --- |
| NM_029084.3, Gene ID: 74748 | atgtggtccctctggagtcttcttctctttgaagctctccttcccgttgtggttgtcagtgtccaagtgctaagcaaggtaggggactcagagctgctggtggccgagtgtcctccgggcttccaagtgcgtgaggctatctggcgatctctgtggccatcggaggagctcctggccacatttttccgaggttccttggagactctgtaccactctcgtttcctgggccgagtccagctatatgacaacctcagcctggagcttggacccctgaaacctggagacagcggcaatttctctgtgctgatggtggatacaaggggtcaaacctggacccagaccctgtatctcaaggtgtacgatgcagtacccaagcccgaggttcaagtgttcactgctgcagcagaggagacccaacccctcaatacctgtcaggtcttcttgtcctgctgggcccccaacatcagtgacataacctacagctggcgacgggaggggacagtggacttcaatggtgaagtgcacagccatttctcaaatggacaggtgttaagtgtctcactgggactgggggacaaggatgtggcctttacctgcattgcctccaatcctgtcagctgggatatgaccacagtcaccccctgggagagctgccatcacgaggcagcctccgggaaggcctcctacaaggacgtgctactggtagtagtgccaattacactgttcctgatcctggctggtctctttggggcatggcaccatggcctctgctcagggaagaagaaggatgcttgcactgacggggtgcttccagagacagagaatgccctcgtatag |

**Table S3 Univariate Cox regression analysis of BCRFS in TCGA dataset**

| **Gene** | **HR** | **HR.95L** | **HR.95H** | **p value** |
| --- | --- | --- | --- | --- |
| *LAIR1* | 1.086 | 1.021 | 1.155 | 0.008 |
| *PTAFR* | 1.048 | 1.010 | 1.088 | 0.012 |
| *HCK* | 1.024 | 1.005 | 1.043 | 0.014 |
| *MYO1F* | 1.061 | 1.012 | 1.112 | 0.015 |
| *NCF4* | 1.029 | 1.004 | 1.054 | 0.020 |
| *HLA-DQA1* | 1.006 | 1.001 | 1.011 | 0.023 |
| *CTSC* | 1.032 | 1.004 | 1.061 | 0.027 |
| *SLAMF8* | 1.029 | 1.001 | 1.056 | 0.039 |
| *ITGB2* | 1.011 | 1.001 | 1.021 | 0.039 |
| *SELPLG* | 1.014 | 1.000 | 1.028 | 0.043 |
| *CIITA* | 1.044 | 1.001 | 1.088 | 0.045 |
| *AIF1* | 1.009 | 1.000 | 1.018 | 0.050 |

**Note: HR, hazard ratio; BCRFS, biochemical recurrence-free survival.**

Table S4 Univariate Cox regression analysis of DMFS in TCGA dataset

| **Gene** | **HR** | **HR.95L** | **HR.95H** | **p** **value** |
| --- | --- | --- | --- | --- |
| *CD86* | 1.199 | 1.100 | 1.306 | 0.000 |
| *LAIR1* | 1.197 | 1.092 | 1.311 | 0.000 |
| *LAPTM5* | 1.008 | 1.004 | 1.013 | 0.001 |
| *IL10RA* | 1.068 | 1.023 | 1.115 | 0.003 |
| *MPEG1* | 1.028 | 1.010 | 1.047 | 0.003 |
| *PTPRC* | 1.026 | 1.009 | 1.044 | 0.004 |
| *CTSC* | 1.066 | 1.021 | 1.113 | 0.004 |
| *APBB1IP* | 1.113 | 1.034 | 1.197 | 0.004 |
| *CD53* | 1.014 | 1.005 | 1.024 | 0.004 |
| *LCP2* | 1.094 | 1.028 | 1.165 | 0.005 |
| *CD4* | 1.022 | 1.006 | 1.038 | 0.006 |
| *ITGB2* | 1.021 | 1.006 | 1.036 | 0.007 |
| *ARHGAP25* | 1.112 | 1.029 | 1.202 | 0.007 |
| *FYB1* | 1.054 | 1.014 | 1.096 | 0.008 |
| *SELPLG* | 1.030 | 1.007 | 1.052 | 0.009 |
| *FERMT3* | 1.034 | 1.008 | 1.061 | 0.010 |
| *CYTH4* | 1.120 | 1.026 | 1.222 | 0.011 |
| *CARD11* | 1.127 | 1.027 | 1.236 | 0.011 |
| *NCKAP1L* | 1.083 | 1.017 | 1.153 | 0.013 |
| *CTSS* | 1.012 | 1.002 | 1.021 | 0.013 |
| *DOK2* | 1.079 | 1.013 | 1.148 | 0.018 |
| *ITGAL* | 1.048 | 1.007 | 1.090 | 0.021 |
| *BIN2* | 1.103 | 1.014 | 1.199 | 0.022 |
| *ARHGAP30* | 1.037 | 1.005 | 1.070 | 0.022 |
| *TNFRSF1B* | 1.023 | 1.003 | 1.044 | 0.023 |
| *SAMSN1* | 1.109 | 1.014 | 1.213 | 0.024 |
| *CYTIP* | 1.049 | 1.006 | 1.094 | 0.025 |
| *SLAMF8* | 1.051 | 1.006 | 1.097 | 0.026 |
| *EVI2A* | 1.075 | 1.009 | 1.146 | 0.026 |
| *CD74* | 1.001 | 1.000 | 1.001 | 0.028 |
| *LSP1* | 1.025 | 1.002 | 1.049 | 0.030 |
| *MYO1F* | 1.096 | 1.007 | 1.193 | 0.035 |
| *SASH3* | 1.024 | 1.001 | 1.047 | 0.037 |
| *NCF4* | 1.047 | 1.002 | 1.095 | 0.042 |
| *HLA-DRB1* | 1.001 | 1.000 | 1.002 | 0.042 |
| *CD52* | 1.006 | 1.000 | 1.013 | 0.045 |
| *HCLS1* | 1.026 | 1.001 | 1.052 | 0.045 |
| *PLEK* | 1.020 | 1.000 | 1.039 | 0.045 |
| *RUNX3* | 1.055 | 1.001 | 1.112 | 0.046 |
| *HCK* | 1.033 | 1.000 | 1.067 | 0.047 |

Note: HR, hazard ratio; DMFS, Distant metastasis-free survival.

**Table S5 Univariate Cox regression analysis of BCRFS in GSE116918 dataset**

| **Gene** | **HR** | **HR.95L** | **HR.95H** | **p value** |
| --- | --- | --- | --- | --- |
| *CD74* | 3.962 | 1.785 | 8.793 | 0.001 |
| *LAPTM5* | 1.874 | 1.289 | 2.726 | 0.001 |
| *CTSC* | 1.685 | 1.202 | 2.363 | 0.002 |
| *DOK2* | 2.716 | 1.319 | 5.591 | 0.007 |
| *TNFRSF1B* | 2.239 | 1.225 | 4.093 | 0.009 |
| *SASH3* | 1.779 | 1.151 | 2.750 | 0.009 |
| *CCL5* | 1.707 | 1.129 | 2.581 | 0.011 |
| *SLAMF8* | 1.440 | 1.077 | 1.926 | 0.014 |
| *MPEG1* | 1.710 | 1.113 | 2.628 | 0.014 |
| *ARHGAP30* | 1.929 | 1.133 | 3.285 | 0.016 |
| *CSF2RB* | 1.358 | 1.053 | 1.751 | 0.018 |
| *CD5* | 2.701 | 1.172 | 6.222 | 0.020 |
| *SLAMF7* | 1.765 | 1.082 | 2.878 | 0.023 |
| *PTPRC* | 1.472 | 1.055 | 2.056 | 0.023 |
| *ITGB2* | 2.013 | 1.082 | 3.742 | 0.027 |
| *PLEK* | 1.364 | 1.035 | 1.798 | 0.028 |
| *ARHGAP25* | 2.383 | 1.095 | 5.183 | 0.029 |
| *TRBC2* | 1.875 | 1.041 | 3.377 | 0.036 |
| *CYTIP* | 1.477 | 1.012 | 2.155 | 0.043 |
| *SELPLG* | 2.119 | 1.011 | 4.441 | 0.047 |

**Note: HR, hazard ratio; BCRFS, biochemical recurrence-free survival.**

**Table S6 Univariate Cox regression analysis of DMFS in GSE116918 dataset**

| **Gene** | **HR** | **HR.95L** | **HR.95H** | **p value** |
| --- | --- | --- | --- | --- |
| *SLAMF8* | 1.735 | 1.108 | 2.715 | 0.016 |
| *PTAFR* | 4.304 | 1.251 | 14.812 | 0.021 |
| *DOK2* | 3.537 | 1.118 | 11.184 | 0.032 |

**Note: HR, hazard ratio; DMFS, Distant metastasis-free survival.**

**Table S7 20 key genes list**

| Gene Name | | | | |
| --- | --- | --- | --- | --- |
| *SAMSN1* | *CD6* | *RUNX3* | *LAIR1* | *SLAMF7* |
| *SLAMF8* | *BIN2* | *CD5* | *EVI2A* | *LSP1* |
| *MPEG1* | *MYO1F* | *ARHGAP9* | *CIITA* | *CXCR3* |
| *RASAL3* | *ARHGAP25* | *NCKAP1L* | *SIT1* | *ARHGAP30* |
